# Molecular prediction for atherogenic risks across different cell types of leukocytes

**DOI:** 10.1186/1755-8794-5-2

**Published:** 2012-01-13

**Authors:** Feng Cheng, Ellen C Keeley, Jae K Lee

**Affiliations:** 1 Department of Biophysics, University of Virginia, Charlottesville, USA; 2 Department of Medicine, Division of Cardiology, University of Virginia, Charlottesville, USA; 3 Department of Public Health Sciences, University of Virginia, Charlottesville, USA

## Abstract

**Background:**

Diagnosing subclinical atherosclerosis is often difficult since patients are asymptomatic. In order to alleviate this limitation, we have developed a molecular prediction technique for predicting patients with atherogenic risks using multi-gene expression biomarkers on leukocytes.

**Methods:**

We first discovered 356 expression biomarkers which showed significant differential expression between genome-wide microarray data of monocytes from patients with familial hyperlipidemia and increased risk of atherosclerosis compared to normal controls. These biomarkers were further triaged with 56 biomarkers known to be directly related to atherogenic risks. We also applied a COXEN algorithm to identify concordantly expressed biomarkers between monocytes and each of three different cell types of leukocytes. We then developed a multi-gene predictor using all or three subsets of these 56 biomarkers on the monocyte patient data. These predictors were then applied to multiple independent patient sets from three cell types of leukocytes (macrophages, circulating T cells, or whole white blood cells) to predict patients with atherogenic risks.

**Results:**

When the 56 predictor was applied to the three patient sets from different cell types of leukocytes, all significantly stratified patients with atherogenic risks from healthy people in these independent cohorts. Concordantly expressed biomarkers identified by the COXEN algorithm provided slightly better prediction results.

**Conclusion:**

These results demonstrated the potential of molecular prediction of atherogenic risks across different cell types of leukocytes.

## Background

Atherosclerosis is the leading cause of death in most countries [1]. In the United States alone, it is estimated that more than 16 million people have atherosclerotic heart disease resulting in 870, 000 deaths in 2005. Diagnosis of subclinical atherosclerosis is difficult since individuals are still asymptomatic. Early detection of atherosclerosis may help to prevent complications of the disease or slow its progression [2]. Atherosclerosis risk can first be indirectly predicted by clinical disease risk factors such as diabetes, LDL, HDL, or triglyceride. The progression of atherosclerosis, however, is a complex multifactorial process which involves multiple molecular mechanisms such as lipid deposition in arteries, smooth muscle cell proliferation, thrombogenesis, and platelet aggregation so it has been found to be difficult to predict atherosclerosis by these standard risk factors alone. For example, Tertov and his collaborators [3] found there was a lack of correlation between the degree of human plasma low density lipoprotein oxidation and its atherogenic potential (r = 0.10, n = 127) and LDL levels showed a weak correlation with a coefficient of 0.12 for total atheroma volume.

Tests based on surgical patient samples such as arterial tissue or atherosclerotic plaques are used to diagnose atherosclerosis but are impractical [4,5]. High throughput molecular techniques have also been used to better understand the pathogenesis and progression of atherosclerosis but have not been fully utilized for diagnostics [4,5]. Invasive or non-invasive imaging methods for plaque in human coronary arteries are currently used to detect the more exact status of atherosclerosis in patients [6,7]. However, use of these imaging methods for plaque detection in human coronary arteries is costly and associated with the risk of adverse events so are often restricted to a small proportion of patients who already show high-risk features and clinical symptoms [7]. Consequently, despite these biotechnical and molecular efforts, diagnosis of subclinical atherosclerosis remains difficult.

Identifying genome-wide biomarkers of various molecular mechanisms relevant to atherogenic risks, we here developed multi-gene prediction signatures for stratifying patients with atherosclerosis using peripheral blood samples. In particular, we addressed two questions in this study: 1) whether subclinical atherosclerosis can be diagnosed by molecular biomarkers in peripheral blood samples, and 2) whether they share certain common molecular expression signatures across different cell types of leukocytes.

## Methods

### Patient and Microarray Data Sets

Four published microarray sets (Table 1) of different leukocyte subsets from atherogenic patients and healthy controls were used to construct and validate our multi-gene predictors. The first set of patients with familial hypercholesterolemia (FH) with microarray data of monocytes, FH1 (NCBI GEO GSE6054), was used as our training set [8]. Familial hypercholesterolemia is a well-known genetic disease caused by a mutation (1 out of 500 people) in the genetic encoding for the LDL receptor (LDLR) gene [9]. This mutation results in dramatically elevated risks of atherosclerosis with most patients experiencing atherogenic complications in their early age. FH patients have thus been frequently followed and investigated for atherosclerosis risks and disease mechanisms. In particular, the risk of atherosclerosis among homozygous FH patients with two abnormal copies of the LDLR gene is increased 5- to 10-fold from that of patients with one abnormal copy or heterozygous FH patients. This set consists of 23 subjects: 3 homozygous FH patients, 7 heterozygous FH patients, and 13 healthy controls.

**Table 1:** Microarray datasets were used for atherosclerosis prediction model derivation and evaluation.

Data Set	Human Blood Cell	Function	GEO ID	Disease information	Samples
FH1	Monocyte	Training	GSE6054	Familial Hypercholesterolemia	23
FH2	Circulating T cells	Test	GSE6088	Familial Hypercholesterolemia	23
FH3	White Blood cells	Test	GSE13985	Familial Hypercholesterolemia	10
ATHERO1	Macrophage	Test	GSE9874	Patient with subclinical atherosclerosis and a family history of coronary heart disease	28

Four previously-published microarray sets in the NCBI GEO database (http://www.ncbi.nlm.nih.gov/geo) from different human blood cells were used to construct and validate our prediction model. Data set FH1 was chosen and used for our multi-gene model development. The other three sets---FH2, FH3, and ATHERO1---were used for evaluation of the atherosclerosis prediction model.

GEO web address: http://www.ncbi.nlm.nih.gov/geo/

Three additional data sets of patients were obtained and used as test sets for our multi-gene predictors. The first test set, FH2 (NCBI GEO GSE6088), was from the same FH patients in the training set but with microarray data of different cell, T-lymphocytes [8]. The second test set, FH3 (NCBI GEO GSE13985), is a completely independent set of 10 FH patients. In this study, whole white blood cells from five FH patients and five controls matched in age, sex, BMI, and smoking status were used for gene expression profiling. All patient samples in FH1, FH2, and FH3 sets were profiled with Affymetrix HG-U133 plus 2.0 GeneChip microarrays.

The third test set, ATHERO1 (NCBI GEO GSE9874), was comprised of 15 patients with asymptomatic atherosclerosis and a family history of coronary heart disease and 15 age- and sex-matched subjects with no atherosclerosis and no family history of coronary heart disease as control [10]. Two arrays were excluded after our initial quality control analysis. Therefore a total of 28 samples was chosen for the model test. The microarrays were obtained from monocyte-derived macrophage cells of these patients' peripheral blood samples with Affymetrix HG-U133A GeneChips. Affymetrix HG-U133A is a part of Affymetrix HG-U133 plus 2.0 so we chose the common probe sets for our model construction and data analysis.

### Development of Multi-Gene Predictors of Atherosclerosis

**Figure 1:**
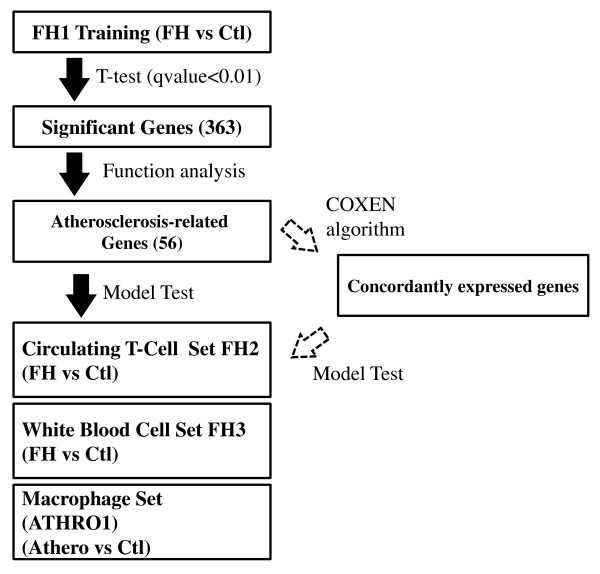
**Schematic diagram of the atherosclerosis prediction model construction and validation processes**. Our prediction model was developed by four distinct steps - 1) the identification of significant genes by t-test analysis of the training set FH1, 2) selected biomarkers known to be directly related to atherosclerosis performed by Ingenuity Pathway Analysis, 3) (optional) selection of COXEN biomarkers among the atherosclerosis-related gene biomarkers obtained from the third step which led to the 48, 6, and 26 biomarkers for test set 1 (circulating T-cell), test set 2 (white blood cell) and test set 3 (macrophage), 4) the multivariate prediction model construction based on universal or COXEN predictors.

Our model training of atherosclerosis predictors was composed of three sequential analysis steps (Figure 1). The first was the identification of significant differentially expressed genes by comparing FH patients and normal controls in the training set by a two sample t-test. In order to avoid the multiple comparisons pitfall in the high throughput biomarker discovery, we adjusted the statistical significance by False Discovery Rate (FDR) q-value < 0.01 [11].

In the second step, we identified a subset of the above biomarkers known to have functions directly related to atherosclerosis and cardiovascular diseases. Biological functional analysis on these genes was performed by the Ingenuity Pathway Analysis (IPA; http://www.ingenuity.com; Redwood City, CA). Genes related to inflammation, lipid metabolism process and another metabolic process (including carbohydrate protein and nucleic acid metabolism) and hematological system developments were selected [12,13].

The last step (Step 3) was the multivariate prediction model construction. Microarray expression values were first standardized within each gene for each set in order to adjust for biased expression distributions of the four independent data sets obtained under different experimental settings. We then applied principal component analysis (PCA) to reduce the high dimension of the multi-gene biomarker space. Top three PCAs were chosen to convey the majority (> 70%) of the information of original genes while their loadings (or weights) reflect the influence of the original genes.

We trained our prediction model using the linear discriminant analysis (LDA) technique, a widely-used multivariate classification technique for our prediction modeling. First, 23 human samples in the training set were divided into a low-risk group (normal controls) and a high-risk group (patients with familial hypercholesterolemia). The LDA is then performed to obtain a linear discriminant function for the two risk classes by maximizing the prediction power or minimizing the overall classification error both for false positives and false negatives with equal weights. The output of the prediction model is the LDA posterior probabilities of atherosclerosis between 0 (low-risk) and 1 (high-risk). Thus, a high predicted score (or posterior probability) of a subject implies a high risk of atherosclerosis whereas a low predicted score suggests a low risk of atherosclerosis.

The performance of prediction models was evaluated to examine if the predicted (posterior) probability of atherosclerosis risk could statistically discriminate patients at high risk for atherosclerosis from normal controls in these three test sets. The statistical significance of the difference in scores was first evaluated using the two-sample t-test between high risk and control groups. To define an optimal cutoff value for each predictor, a Receiver Operating Characteristic (ROC) curve was plotted for the predicted scores of atherosclerosis risk and phenotype information on each set. The cutoff value on each ROC curve was then determined by maximizing the Youden index (= sensitivity+specificity-1) [14]. Sensitivity, specificity, positive predictive value, (PPV), and negative predictive value (NPV) were then calculated to evaluate its prediction performance at the optimal cutoff values.

Sensitivity, specificity, PPV, NPV were defined as

$$Sensitivity=\frac{\text{number}\;\text{of}\;\text{true}\;\text{positives}}{\text{number}\;\text{of}\;\text{true}\;\text{positives}\;\text{ + }\;\text{number}\;\text{of}\;\text{false}\;\text{negatives}}$$

$$Specificity=\frac{\text{number}\;\text{of}\;\text{true}\;\text{negatives}}{\text{number}\;\text{of}\;\text{true}\;\text{negatives}\;\text{ + }\;\text{number}\;\text{of}\;\text{false}\;\text{positives}}$$

$$PPV=\frac{\text{number}\;\text{of}\;\text{true}\;\text{positives}}{\text{number}\;\text{of}\;\text{true}\;\text{positives}\;\text{ + }\;\text{number}\;\text{of}\;\text{false}\;\text{positives}}$$

$$NPV=\frac{\text{number}\;\text{of}\;\text{true}\;\text{negatives}}{\text{number}\;\text{of}\;\text{true}\;\text{negatives}\;\text{ + }\;\text{number}\;\text{of}\;\text{false}\;\text{negatives}}$$

**Figure 2:**
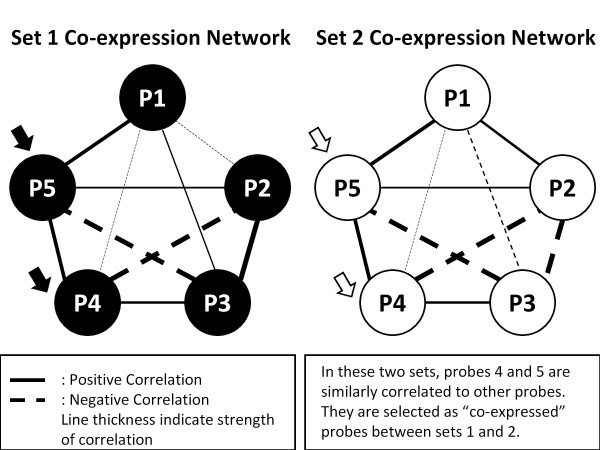
**A schematic illustration of COXEN algorithm by using an artificial five-probe example**. Probes 1 and 3 in cell set show essentially the same patterns as probes 1 and 3 in cell set 2. These two probes have the same co-expression correlation with other probes in these two cell sets. On the contrary, probes 2, 4, and 5 show different patterns of co-expression correlation in these two cell sets. Therefore, Probes 1 and 3 (but not 2, 4, and 5) will be selected by the COXEN algorithm.

We also applied the COXEN algorithm, a previously published bioinformatics technique that can identify concordantly (expression) regulated genes between different cancer types [15,16] to improve our prediction on patient sets from different cell types of leukocytes. White blood cells (WBC) are a heterogeneous mixture of many different cell types, each of which can present some different expression patterns [8]. Here, we applied this technique to identify concordantly expressed biomarkers between different types of leukocytes. The COXEN algorithm compared expression patterns among candidate genes between the two sets to find concordantly expressed genes between them. Figure 2 is a schematic illustration of the COXEN algorithm by using an artificial 5-gene probe example [15]. In this figure, gene probes 1 and 3 in cell set 1 (e.g., the monocyte in this paper) essentially show the same patterns as probes 1 and 3 in cell set 2 (e.g., the T-lymphocyte). That is to say, these two probes have the same co-expression correlation with other probes in these two cell sets. This co-expression correlation is analyzed by Spearman correlation. On the contrary, probes 2, 4, and 5 show different patterns of co-expression correlation in these two cell sets. In this example, gene probes 1 and 3 (but not 2, 4, and 5) are selected by the COXEN algorithm. More technical details of the COXEN analysis can be found elsewhere [15,16]. By using the COXEN algorithm to the above monocyte-derived biomarkers (identified in Step 2), we identified three subsets of genes from these 56 genes which showed concordant expression network patterns between monocyte and each of T-lymphocyte, macrophage, or whole WBC, respectively. We also tested the predictive performance of these COXEN genes in this paper.

## Results

### Identification of atherosclerosis-associated biomarkers

The initial biomarker discovery step (Step 1) identified 363 differentially expressed genes by comparing FH patients and normal controls in the training set FH1 with q-value < 0.01 (Figure 1). The second function analysis was performed by identifying genes of which biological functions might be closely related to atherosclerosis using the Ingenuity Pathway Analysis (IPA) software (Redwood City, CA). In particular, Genes related to inflammation, lipid metabolism process and another metabolic process and hematological system developments were selected. This functional analysis was necessary since differential expression of some of the initial biomarkers might not be directly relevant to the atherogenic disease mechanisms and only specific to the training patient set FH1 we used. This step resulted in 56 gene probes whose functional information is summarized in Table 2.

**Table 2:** Selected atherosclerosis related genes for model derivation.

Probes	Gene_Symbol	Pvalue	Qvalue	Fold change	Predictor for test set	Function
211921_x_at	PTMA	8.88E-08	5.79E-04	1.47	MO/MA	Inflammatory Disease
206788_s_at	CBFB	2.87E-07	7.32E-04	-1.50	MO/MA	Inflammatory Response
207760_s_at	NCOR2	2.49E-07	7.32E-04	1.25	MA	Other Metabolic Processes
218828_at	PLSCR3	2.05E-07	7.32E-04	1.27	MO/WBC	Other Metabolic Processes
201331_s_at	STAT6	2.74E-06	2.18E-03	1.32	MO	Inflammatory Response
201591_s_at	NISCH	2.55E-06	2.18E-03	1.28	MO/WBC	Other Metabolic Processes
205125_at	PLCD1	2.19E-06	2.18E-03	1.22	MO	Other Metabolic Processes
202102_s_at	BRD4	3.25E-06	2.39E-03	1.41	MO/WBC	Other Metabolic Processes
205059_s_at	IDUA	3.94E-06	2.44E-03	1.38	MO/MA	Other Metabolic Processes
212705_x_at	PNPLA2	4.25E-06	2.56E-03	1.36	MO/MA	Lipid Metabolism
203839_s_at	TNK2	4.95E-06	2.88E-03	1.37	MO/MA	Other Metabolic Processes
210969_at	PKN2	5.25E-06	2.95E-03	-1.55	MO	Other Metabolic Processes
207292_s_at	MAPK7	5.67E-06	2.97E-03	1.23	MO	Other Metabolic Processes
211716_x_at	ARHGDIA	5.74E-06	2.97E-03	1.43	MO	Inflammatory Disease
206580_s_at	EFEMP2	6.76E-06	3.24E-03	1.35	MO	Inflammatory Disease
202389_s_at	HTT	7.10E-06	3.25E-03	1.17	WBC	Lipid Metabolism
218961_s_at	PNKP	7.00E-06	3.25E-03	1.22	MO	Other Metabolic Processes
1555214_a_at	CLEC7A	7.61E-06	3.44E-03	-1.89	MO	Inflammatory Response
220088_at	C5AR1	8.96E-06	3.56E-03	1.32	MO	Inflammatory Response
204506_at	PPP3R1 /WDR92	1.01E-05	3.87E-03	-1.51	MO/MA	Other Metabolic Processes
202848_s_at	GRK6	1.25E-05	4.08E-03	1.13	MO	Inflammatory Response
204524_at	PDPK1	1.42E-05	4.25E-03	1.36	MO	Other Metabolic Processes
211537_x_at	MAP3K7	1.55E-05	4.25E-03	-1.28	MO/MA	Inflammatory Response
224883_at	PLDN	1.57E-05	4.25E-03	-1.27	MO	Inflammatory Response
205926_at	IL27RA	1.70E-05	4.30E-03	1.75	MO/MA	Inflammatory Response
202264_s_at	TOMM40	1.90E-05	4.51E-03	1.24	MO/MA	Inflammatory Response
210995_s_at	TRIM23	1.91E-05	4.51E-03	-1.67	MO/MA	Other Metabolic Processes
210809_s_at	POSTN	1.98E-05	4.60E-03	1.19	MO	Inflammatory Disease
207904_s_at	LNPEP	2.08E-05	4.73E-03	-1.60	MO/MA	Other Metabolic Processes
226111_s_at	ZNF385A	2.29E-05	4.95E-03	1.40	MO	Hematological System Development and Function
205400_at	WAS	2.68E-05	5.31E-03	1.48	MO/MA	Lipid Metabolism
203709_at	PHKG2	2.87E-05	5.57E-03	1.21	MA	Other Metabolic Processes
209812_x_at	CASP2	3.02E-05	5.72E-03	-1.26	MO	Lipid Metabolism
221957_at	PDK3	3.48E-05	6.13E-03	-1.34	MO/MA	Other Metabolic Processes
1557145_at	STK38	3.61E-05	6.15E-03	-1.50		Other Metabolic Processes
217888_s_at	ARFGAP1	3.69E-05	6.18E-03	1.19	MO	Inflammatory Disease
204158_s_at	TCIRG1	3.98E-05	6.35E-03	1.28		Immunological Disease
204150_at	STAB1	4.16E-05	6.47E-03	1.46		Inflammatory Response
36936_at	TSTA3	4.17E-05	6.47E-03	1.16	MO/MA	Carbohydrate Metabolism
211652_s_at	LBP	4.51E-05	6.57E-03	1.20	MO	Lipid Metabolism
225647_s_at	CTSC	4.74E-05	6.80E-03	-1.36	MO	Inflammatory Disease
200604_s_at	PRKAR1A	6.14E-05	7.77E-03	-1.54	MO/MA	Other Metabolic Processes
202804_at	ABCC1	6.52E-05	7.80E-03	1.15	MO	Lipid Metabolism
221563_at	DUSP10	6.60E-05	7.82E-03	-1.26	MO	Other Metabolic Processes
207240_s_at	LHCGR	7.14E-05	8.15E-03	1.09		Carbohydrate Metabolism
206217_at	EDA	7.32E-05	8.21E-03	1.32	MO/MA	Other Metabolic Processes
208867_s_at	CSNK1A1	7.69E-05	8.34E-03	-1.39	MA	Other Metabolic Processes
221770_at	RPE	8.05E-05	8.40E-03	-1.29	MO/WBC/MA	Carbohydrate Metabolism
207319_s_at	CDC2L5	8.46E-05	8.70E-03	-1.59	MO/MA	Hematological System Development and Function
207764_s_at	HIPK3	9.28E-05	9.18E-03	-1.57	MO/MA	Other Metabolic Processes
209532_at	PLAA	9.57E-05	9.33E-03	-1.19	MO	Lipid Metabolism
210333_at	NR5A1	9.76E-05	9.43E-03	1.37	MO/WBC/MA	Hematological System Development and Function
205546_s_at	TYK2	1.02E-04	9.67E-03	1.16	MO	Inflammatory Response
207201_s_at	SLC22A1	1.10E-04	9.97E-03	1.33	MO/MA	Other Metabolic Processes
213733_at	MYO1F	1.11E-04	1.00E-02	1.15		Inflammatory Response
214971_s_at	ST6GAL1	1.11E-04	1.00E-02	-1.46	MO/MA	Inflammatory Response

These genes were identified by t-test, biological function analysis and COXEN algorithm

MO: Monocyte (Test set1); WBC: White blood cell (Test set2); MA: Macrophage (Test set3)

### Independent Evaluation of Atherosclerosis Risk Prediction for Test Sets

**Figure 3:**
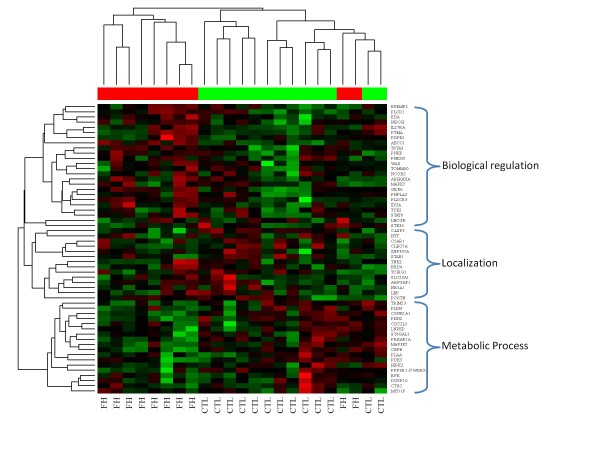
**Clustering Analysis of 56 genetic biomarkers for test set FH2**. Test set FH2 was standardized (subtracted by its mean and divided by its standard deviation) for each gene and a hierarchical clustering analysis was performed on this set. McQuitty's or WPGMA method, which uses the average distance between clusters weighted by uneven cluster sizes, was used. Three functional subclusters of the 56 biomarkers were identified by examining common biological functions of these gene subclusters by the function annotation tool in DAVID database (http://david.abcc.ncifcrf.gov).

Broad predictive information of these genes could be found in a standard hierarchical clustering analysis using the so-called McQuitty's or WPGMA algorithm which is known to be robust with highly varying sizes of clusters by using the average distance between clusters weighted by uneven cluster sizes. We performed a clustering analysis of the 56 biomarkers on one test dataset---FH2---by standardizing (subtracted by its mean and divided by its standard deviation) each gene's expression values across all patient samples (Figure 3). This clustering heatmap showed that all but two FH patients clustered into their respective groups. These 56 genes were also found to be well clustered into three sub-functional categories: biological regulation, localization, and metabolic process.

**Figure 4:**
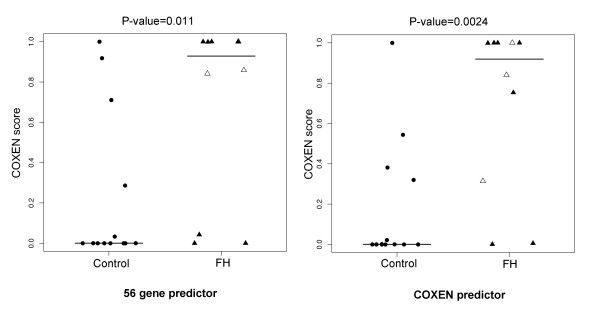
**Prediction results of the data set FH2 using universal and COXEN predictors**. Comparison of LDA probabilities of Familial Hypercholesterolemia (FH) patients and control people in data set FH2. Normal controls, homozygous and heterozygous FH patients are labeled with dots, empty and filled triangles respectively. The statistical significance (p-value) of the set of predictions was assessed by a two sample t-test.

We independently evaluated the performance of our 56-gene predictor of T-cells on the FH2 set (Table 3). To examine the distributional differences of the predicted scores among 3 homozygous FH patients, 7 heterozygous FH patients and 13 normal controls in FH2 data set, the predicted scores of the two groups were plotted (Figure 4). We also compared predicted scores between the high-risk group (FH patients) and low-risk group (controls) by the Student's two-sample t-test. The predicted scores of the control (low-risk) group were significantly lower than those of the FH patient (high-risk) group (p = 0.01). Furthermore, the predicted scores of homozygous FH patients with a higher risk of atherosclerosis were found to be considerably higher than those of heterozygote FH patients who generally show a lower risk of atherosclerosis. The high- and low-risk groups could be well distinguished at the optimal cutoff value in the ROC analysis by maximizing the Youden index (with sensitivity = 70.0% and specificity = 92.3%). Thus, the 56-gene COXEN predictor for T-lymphocytes significantly stratified the two different risk groups of atherosclerosis.

**Table 3:** Prediction Results for three test sets.

	No. of genes for prediction	T-test (P-value)	Sensitivity (%)	Specificity (%)	PPV (%)	NPV (%)	Youden index
56 gene model							

FH2	56	0.011	70.0	84.6	77.8	78.6	0.55
FH3	56	0.010	80.0	100	100	0.83	0.8
ATHRO1	56	0.019	71.4	71.4	71.4	71.4	0.43
							

COXEN model							

	48	0.0024	70.0	92.3	87.5	80.0	0.62
	6	1.5e-10	100	100.0	100.0	100	1.00
	25	0.010	78.6	66.7	68.8	76.9	0.45

Data sets FH2, FH3, and ATHERO1 were used to evaluate the classification performance of our atherosclerosis prediction model. We tested if the predicted risk of atherosclerosis could statistically discriminate familial hypercholesterolemia or general atherosclerosis patients with normal people in these test sets. The statistical significance of the difference in predicted scores (COXEN SCORE) was evaluated using the two-sample t-test between patients and normal groups. Sensitivity, specificity, positive predictive value (PPV), and negative predictive value (NPV) were also calculated.

**Figure 5:**
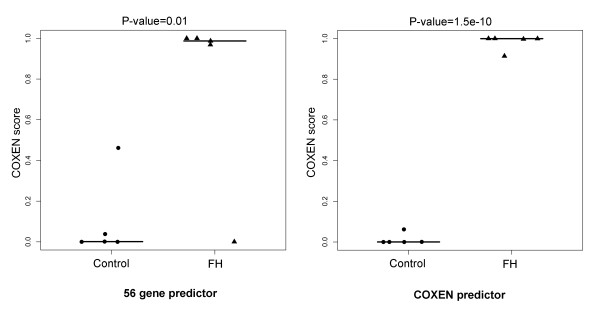
**Prediction results of the data set FH3 using universal and COXEN predictors**. Comparison of LDA probabilities of FH patients and healthy controls in FH3. The statistical significance (p-value) of the set of predictions was assessed by a two sample t-test.

In the next application, we used another independent patient dataset FH3 of total white blood cells to test the predictability of the same 56-gene model for WBC. This small cohort consisted of 5 FH patient and 5 healthy controls. As shown in Figure 5, this 56-gene predictor was also able to significantly discriminate the two groups showing predicted scores of the FH patients significantly higher than those of the controls (two sample t-test p-value = 0.01, Table 3) with perfect classification specificity = 1 and sensitivity = 1 at the Youden cutoff value for this small set.

We next applied the identical 56-gene predictor to the ATHERO1 set of asymptomatic atherosclerosis patients. This set included 28 blood macrophage samples from 14 patients confirmed with asymptotic atherosclerosis and 14 healthy controls, both groups with a family history of coronary artery disease. In particular, 14 patients were diagnosed with atherosclerosis by CTA but they had not shown any clinical symptoms with little clinical difference from the control subjects with a family history of CAD. Therefore, this stratification was challenging and we believe it could be similar to difficult cases of diagnosis in clinics.

**Figure 6:**
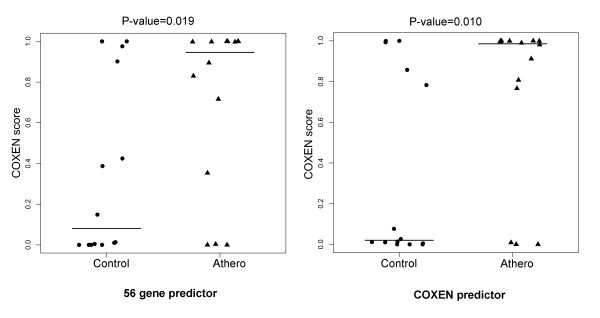
**Prediction results of the data set ATHERO1 using universal and COXEN predictors**. Comparison of LDA probabilities of patient with subclinical atherosclerosis and a family history of coronary heart disease (CHD) and the age and sex-matched subjects with no atherosclerosis and no family history of CHD (control group). The statistical significance (p-value) of the set of predictions was assessed by a two sample t-test.

Using the prediction model of these 56 biomarkers on FH1, we tested this multi-gene predictor on macrophage cell data for predicting the risk of asymptomatic atherosclerosis. As shown in Figure 6, the predicted scores of asymptomatic atherosclerosis patients were significantly higher than those of the controls (t-test p-value = 0.02, Table 3). This result was again quite encouraging indicating that our multi-gene model could provide the predictability for asymptomatic atherosclerosis patients.

### COXEN applications

While our universal 56-gene model showed consistent prediction performance on the three independent test sets, we further investigated whether these predictions could be improved by using biomarkers that preserved highly concordant expression patterns between different cell types of leukocytes. In particular, we used the COXEN algorithm originally developed to discover concordant expression biomarkers between cancer cell lines and human patients with cancer. Applying the COXEN algorithm, we identified 48, 6, and 25 genes (among these 56 genes) that preserved highly concordant gene expression patterns between patient sets FH1 and FH2, FH1 and FH3, or FH1 and ATHERO1, respectively, with a Pearson correlation association p-value < 0.001 (Table 2).

The number of COXEN genes generally reflects the similarities between two data sets. FH1 and FH2 were found to share the most concordantly expressed genes (48 genes among 56) since these two data sets were obtained from the same set of patients. For the other two data sets, the ATHERO1 set from macrophages was found to result in more COXEN genes (25 genes among 56) that were concordantly expressed with those of the FH1 monocyte set than the WBC FH3 set (6 among 56). Thus, macrophage cells appeared to preserve many common gene expression patterns with monocyte cells. On the contrary, WBC, which is a mixture of different cell types, showed less consistent gene expression patterns with monocytes than the other two sets. As shown in Table 3 these COXEN predictors provided even better prediction results. The three COXEN biomarker predictors showed very significant t-test p-values for stratifying high risk patients from controls to 0.0024, 1.5e-10, and 0.01, respectively. Thus, the COXEN predictors showed considerable improvement from the universal prediction model.

Gene function analysis by the IPA software (Redwood City, CA) showed 6 of 48 biomarkers of T-lymphocyte cells were directly related to leukocyte activation and a change in morphology and behavior of a leukocyte including lipopolysaccharide binding protein (LBP), interleukin 27 receptor, alpha (IL27RA), mitogen-activated protein kinase kinase kinase 7 (MAP3K7), pallidin homolog (PLDN), signal transducer and activator of transcription 6, interleukin-4 induced (STAT6) and C-type lectin domain family 7, member A (CLEC7A). This clearly implied that T-lymphocyte activation was involved in atherogenesis. 6 genes selected for white blood cell FH3 prediction were phospholipid scramblase 3 (PLSCR3), nischarin (NISCH), bromodomain containing 4 (BRD4), huntingtin (HTT), ribulose-5-phosphate-3-epimerase (RPE) and nuclear receptor subfamily 5, group A, member 1 (NR5A1). Also, the 6 biomarkers were the phosphoproteins of which phosphate groups regulate protein functions by esterifying to serine, threonine or tyrosine. In particular, NR5A1 is known to be a nuclear receptor that regulates the transcription of many genes involved in atherogenesis and PLSCR3 has been proved to be related to atherosclerosis development by animal studies [17].

## Discussion

In this study, we developed multi-gene biomarker models to predict early-stage (asymptomatic) atherosclerosis based on different types of leukocytes. In particular, we have shown a proof-of-concept strategy to predict asymptomatic atherosclerosis by using molecular biomarkers from peripheral blood samples, and the existence of common molecular expression signatures of atherogenic risks across different cell types of WBC. We believe these predictions were possible due to several reasons. First, gene expression signatures from patient blood samples appear to have a high potential to predict atherosclerosis in its early stage since certain molecular changes in blood cells may occur much before the plaque development or serious clinical symptoms. Second, a genome-wide microarray technique enabled us to comprehensibly identify relevant molecular expression signatures of atherogenic risks beyond the information obtained from standard clinical parameters. We also believe our multi-gene predictors could predict the risk of atherosclerosis more accurately than predictors based on single biomarkers or a small number of clinical parameters. Finally, we found many biomarkers of atherosclerosis shared consistent expression patterns across different leukocyte subsets. It will be interesting to further investigate these common biomarkers for their specific roles in the disease.

We note that FH1 and FH2 were from the identical set of familial hyperglycemia patients and healthy controls, and possibly that our significant prediction on FH2 is correlated with the use of the identical patient set. However, we believe that our significant prediction on FH2 is not mainly due to the use of the same patient set for the following reasons. First, the molecular data of the two sets were from completely different immune cells---FH1 from monocytes and FH2 from circulating T cells. Since our biomarker discovery and predictive modeling were performed strictly based on monocyte cells of the FH1 set, FH2 is independent of FH1 for its molecular characteristics and data. Second, we observed that our identical prediction model performed considerably better for white blood samples on FH3, a completely independent set of FH patients and controls from the FH1 set. We think this was due to the fact that monocytes are partially included in white blood cells so our monocyte-based predictor presented a better predictability for that set. Therefore, the common molecular information appears to be more important than the use of specific patient set for our training and prediction.

We believe our approach can be highly useful for clinical diagnosis of atherosclerosis for the following reasons. First, we used molecular signatures from patients' blood samples that can be conveniently obtained in routine clinical practice. Second, we found that molecular signatures of atherogenic risks exist and are commonly shared among different types of leukocytes so we may be able to choose and further refine diagnosis tests based on one or multiple types depending on their accuracy and clinical applicability. For example, clinical data showed that monocyte cells are involved in very early pathogenetic stages of atherosclerosis [18]. In particular, when atherosclerosis plaques (or foam cells) are differentiated from monocytes recruited from circulating blood, critical molecular changes appear to occur much before any clinical symptoms of atherosclerosis. Thus, a molecular test based on blood monocyte cells may serve as an effective diagnosis tool for an early stage of atherosclerosis. Also, as seen in several patient data sets we investigated here, microarray profiling of a small amount of blood cells can now be efficiently and cost-effectively performed so its use becomes quite practical for clinical applications as well as scientific investigations. However, once final biomarkers and prediction models are identified and finalized, diagnosis tests and assays can also be developed with more economic and convenient techniques such as RT-PCR.

There are several limitations in our current study. First, our primary biomarker discovery and prediction model training were performed by contrasting familial hypercholesterolemia patients against healthy controls. Likely due to this restriction, our prediction was better in stratifying FH patients from healthy controls than general subclinical atherosclerosis patients. When we reversed the role of training and test sets in our preliminary analysis, i.e. used the subclinical atherosclerosis patient set for model training and FH patient sets for independent model test, the prediction results were generally deteriorated, possibly due to the small sample size of the subclinical atherosclerosis patient set (data not shown). Questions regarding whether predictors can perform significantly better if they are trained based on the same disease type of patients and/or same subtype of blood cells requires more careful investigation with a larger number of patient data in a future study. Also, we found that COXEN biomarker discovery and modeling training based on monocyte data was more successful to predict risks based on other cell types than other directions, e.g. T-cell data for training to predict the others. It may be due to the data quality or biological information in the monocyte data which requires further investigation.

Even though we did not use patients' outcome information in our COXEN-based predictions, we partially used the molecular information of our test patient data sets in the current study. A more rigorous prediction performance of these predictors should thus be further evaluated using a third patient set from the same cell type of leukocytes. Our current predictors were constructed solely based on molecular data due to the lack of patients' other clinical information in our datasets. However, we believe additional predictive information can be obtained from many clinical parameters of patients such as age, gender [19], LDL level, HDL level, apolipoproteins or triglyceride level. Also one of the keys to enhancing the success rate by the prediction model in the future is that "six stages of atherosclerotic lesion" were used to construct the model, rather than only using "0" or "1" to represent the atherogenic risk of the atherosclerosis patients. If relevant clinical data are available for our model development, we believe prediction of atherogenic risks can be further improved by constructing models both with molecular and clinical parameters.

## Conclusion

We discovered 56 atherosclerosis-related expression biomarkers which showed significant differential expression between genome-wide microarray data of monocytes from patients with familial hyperlipidemia and increased risk of atherosclerosis compared to normal controls. Based on these biomarkers, we developed our multi-gene biomarker model that could predict early-stage (asymptomatic) atherosclerosis across different types of leukocytes. We also applied a COXEN algorithm to identify concordantly expressed biomarkers between monocytes and each of three different cell types of leukocytes---macrophage, circulating T, and whole white blood cells. We then developed three separate multi-gene predictors using the three subsets of these 56 biomarkers using the monocyte patient data as the training set. These individual predictors showed further improved statistical power for predicting early-stage atherosclerosis. We thus believe these predictors can be quite useful for developing diagnostic tools for patients who are at an increased risk of cardiovascular disease.

## Competing interests' statement

The authors declare that they have no competing interests.

## Authors' contributions

FC designed the study, conducted data acquisition, analysis, and wrote the manuscript. EK provided clinical input and investigation directions for atherosclerosis prediction and wrote the manuscript. JL designed the study, guided the analysis, and wrote the manuscript. All authors have read and approved the final manuscript.

## Pre-publication history

The pre-publication history for this paper can be accessed here:

http://www.biomedcentral.com/1755-8794/5/2/prepub
